# Nanosized Delivery Systems for Therapeutic Proteins: Clinically Validated Technologies and Advanced Development Strategies

**DOI:** 10.3389/fbioe.2020.00089

**Published:** 2020-02-14

**Authors:** Filippo Moncalvo, Maria Isabel Martinez Espinoza, Francesco Cellesi

**Affiliations:** Dipartimento di Chimica, Materiali e Ingegneria Chimica “G. Natta”, Politecnico di Milano, Milan, Italy

**Keywords:** therapeutic proteins, protein delivery, polymer conjugates, PEGylation, liposomes, nanocarriers

## Abstract

The impact of protein therapeutics in healthcare is steadily increasing, due to advancements in the field of biotechnology and a deeper understanding of several pathologies. However, their safety and efficacy are often limited by instability, short half-life and immunogenicity. Nanodelivery systems are currently being investigated for overcoming these limitations and include covalent attachment of biocompatible polymers (PEG and other synthetic or naturally derived macromolecules) as well as protein nanoencapsulation in colloidal systems (liposomes and other lipid or polymeric nanocarriers). Such strategies have the potential to develop next-generation protein therapeutics. Herein, we review recent research progresses on these nanodelivery approaches, as well as future directions and challenges.

## Introduction

Over the last few years, several therapeutic proteins have been approved for clinical usage, and others are in the process of development (Leader et al., 2008; Walsh, 2018). Nowadays, approximately 40% of the 6,000 or more products wordwide currently in clinical development are biopharmaceuticals, in which the predominance of protein-based products is likely to remain an industry reality for the next years (Walsh, 2018).

From a therapeutic perspective, the success of therapeutic protein products is related to their increased specificity and high potency, longer duration of their effect due to the slower clearance from the body, and reduced intrinsic toxicity (Yin et al., 2015). These characteristics provide a clear advantage over low molecular weight drugs, which are generally associated with off-target effects and harmful metabolites. With the use of recombinant DNA technology, therapeutic proteins have been developed to treat a wide variety of disease, including cancers, autoimmunity/inflammation, exposure to infectious agents, and genetic disorders (Leader et al., 2008).

Despite these advantages, these products must overcome the typical drawbacks of short half-life, instability, and immunogenicity, and limited permeability through the biological barriers, due to their high molecular weight (Kintzing et al., 2016). Several strategies have been evaluated in order to improve these limitations and develop a next generation protein therapeutics (Kintzing et al., 2016; Lagassé et al., 2017).

Most efforts have been devoted to the modification of the protein structure, either by mutation or by covalent attachment of moieties, including Fc-fusion (Levin et al., 2015), albumin-fusion (Lagassé et al., 2017), synthetic polypeptide (XTEN) fusion (Schellenberger et al., 2009), the conjugation of polymers such as poly(ethylene glycol) (PEG) or alternative non-degradable/biodegradable macromolecules. A change in drug formulation, introducing liposomes and other lipid-based or polymeric nanocarriers, has also been used to overcome the current limitations of protein therapeutics.

The intent of this review is to highlight the recent advances in developing nanosized delivery systems to improve safety and efficacy of protein therapeutics. This includes the areas of polymer conjugates (such as PEGylation and more recent technologies), liposomes, as well as alternative strategies based on protein nanoencapsulation in lipid-based and polymer-based nanocarriers. The advantages and limitations of systems that have reached the clinical stage are discussed, and advanced delivery strategies are also examined, aiming to provide useful insights for future development.

## Protein-Polymer Conjugates

Protein-polymer conjugates are widely used as therapeutics, since these nanosystems display a unique combination of properties derived from both materials (i.e., the protein and the polymer), which can be individually tuned to obtain the desired effects (Pelegri-O’Day et al., 2014). Polymer conjugates that display enhanced pharmacokinetic properties along with improved stability and/or degradability will be presented hereafter.

### PEGylation

Poly(ethylene glycol) (PEG) is a synthetic, hydrophilic and FDA-approved polymer, typically synthesized using a ring-opening polymerization of ethylene oxide to produce a broad range of polymers with targeted molecular weight, narrow molecular weight distribution, and desired terminal functional groups (Zalipsky, 1995). Due to its biocompatibility and protein-repellent properties, PEG is frequently used in many biomedical applications including bioconjugation and drug delivery (Veronese and Pasut, 2005; Cheng et al., 2007; Bruni et al., 2017). Bioconjugation with PEG, also known as PEGylation, is the formation of a covalent bond between therapeutic molecules and PEG in order to extend circulation half-life of therapeutics, thus reducing the frequency of dosing while maintaining the pharmaceutical effects (Grigoletto et al., 2016).

PEG is well-known as “stealth” molecule; due to its protein-repellent properties, it exhibits low opsonization, and this allows PEG conjugates to avoid phagocytosis and fast removal from the bloodstream (Owens and Peppas, 2006). Additionally, PEGylation also limits the interaction with enzymes, thus inhibiting the breakdown of the therapeutic (bio)molecules *in vivo* (Harris and Chess, 2003).

In case of small proteins or peptides, the right choice of PEG molecular weight may further prolong the circulation time of the biomolecules by enhancing their hydrodynamic radii, up to a size which prevents excretion through the kidney filtration barrier (Xue et al., 2013). Narrow molecular weight distributions (low dispersity) are generally favored for approval by the regulatory authorities, as they guarantee uniformity in the final physico-chemical properties of the product (Jevsevar et al., 2010). In some cases, polymer branching may also be useful in reducing the viscosity of the protein suspension to be injected, and mimicking the glycosylation patterns on native proteins (Pelegri-O’Day et al., 2014). Since the first PEGylated protein approved by the FDA in 1990, PEG bioconjugation has been extensively used for proteins modification, leading to several PEGylated-proteins approved for clinical use (Table 1).

**TABLE 1:** List of approved PEGylated proteins of therapeutic use.

**Generic name**	**Brand name**	**PEGylated protein**	**PEGylation**	**Therapeutic indication**	**Year**	**References**
**(A) Proteins with non-specific PEGylation.**						
Pegadamase	Adagen^®^	Bovine adenosine deaminase	Random amine PEGylation multiple linear 5 kDa PEG	Severe combined immunodeficiency disease	1990	Levy et al., 1988
Pegaspargase	Oncaspar^®^	L-asparaginase	Random amine PEGylation multiple linear 5 kDa PEG	Acute lymphoblastic leukemia	1994	Graham, 2003
Peginterferon-α2b	PegIntron^®^	IFN-α2b	Random amine PEGylation linear 12 kDa PEG	Hepatitis C	2000	Wang et al., 2002
Peginterferon-α2a	Pegasys^®^	IFN-α2a	Random amine PEGylation branched 40 kDa PEG	Hepatitis C	2001	Foser et al., 2003
Pegvisomant	Somavert^®^	Genetically engineered hGH	Random amine PEGylation multiple linear 5 kDa PEG	Acromegaly	2002	Pradhananga et al., 2002
CERA	Mircera^®^	Epoetin-β	Random amine PEGylation linear 30 kDa PEG	Anemia associated with kidney disease	2007	Macdougall and Eckardt, 2006
Pegloticase	Krystexxa^®^	Uricase	Random amine PEGylation 10 kDa PEG	Chronic gout	2010	Schlesinger et al., 2010
Peginterferon-α2b	Sylatron	INF-α2b	Random PEGylation at different site with linear 12 kDa PEG	Melanoma	2011	Patel and Walko, 2012
Rurioctocog alfa pegol	Adynovi^®^/Adynovate^®^	Coagulation factor VIII	Random amine PEGylation branched 20 kDa PEG	Hemophilia A	2015	Dunn et al., 2018
Pegvaliase	Palynziq^®^	Phenylalanine ammonia lyase	Random amine PEGylation 20 kDa PEG	Phenylketonuria	2018	Levy et al., 2018
**(B) Site-directed PEGylated products.**						
Pegfilgrastim	Neulasta^®^	G-CSF	N-terminal PEGylation linear 20kDa PEG	Neutropenia during chemotherapy	2002	Piedmonte and Treuheit, 2008
Certolizumab Pegol	Cimzia^®^	Fab’ antibody fragment	Site specific thiol PEGylation branched 40 kDa PEG	Rheumatoid arthritis and Crohn’s disease	2008	Blick and Curran, 2007
Lipegfilgrastim	Lonquez^®^	G-CSF	Site specific single 20-kDa via carbohydrate linker	Neutropenia	2013	Mahlert et al., 2013
Peginterferon-β1a	Plegridy^®^	INF-β1a	N-terminal PEGylation Linear 20 kDa PEG	Multiple sclerosis	2014	Chaplin and Gnanapavan, 2015
Nonacog beta pegol	Refixia^®^	Coagulation factor IX	A 40 kDa PEG attached to the FIX activation peptide by site-directed glycoPEGylation	Hemophilia B	2017	Ezban et al., 2019
Damoctocog alfa pegol	Jivi^®^	Coagulation factor VIII	Site specific 60 kDa branched PEG (two 30 kDa chains)	Hemophilia A	2018	Paik and Deeks, 2019
Turoctocog alfa pegol	Esperoct^®^	Coagulation factor VIII	40 kDa PEG bound by a unique O-linked glycan on the residual 21 amino acid B-domain region	Hemophilia A	2019	Novo-Nordisk, 2019

### Conjugation Strategies

**FIGURE 1:**
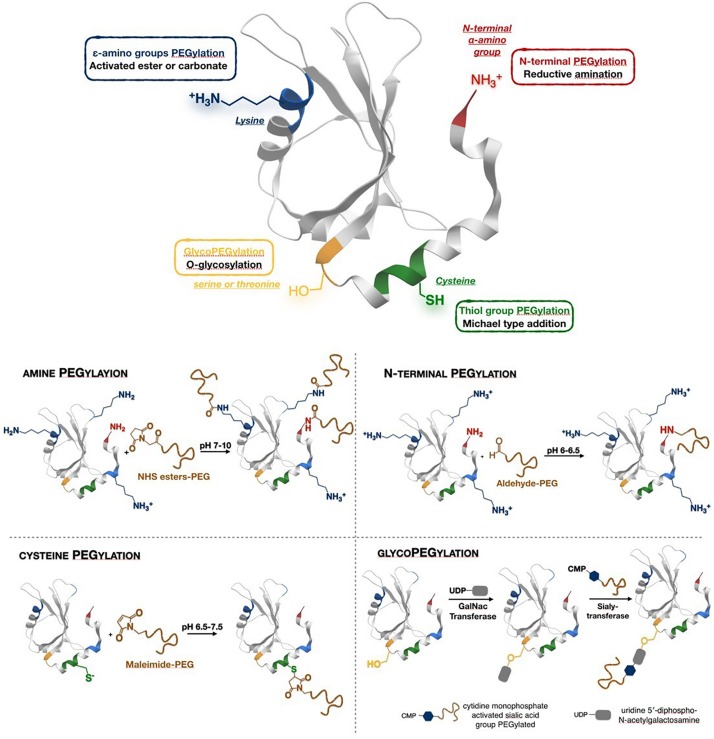
Different conjugation strategies used for protein PEGylation.

PEG reagents are functionalised PEG-based polymers which allow stable bond formation with specific functional groups from the amino acid sequence of the protein. Different sites can be targeted for PEGylation (Figure 1). Many PEG functionalised with activated esters [succinimidyl succinate (PEG-SS), *N*-hydroxysuccinimide esters (PEG-NHS)] and carbonates (PEG p-nitrophenyl carbonate) target the ε-amino groups of lysines, due to their abundance on the protein surface. This conjugation is generally non-selective, and other groups (N-terminal amines, histidine, tyrosine) can also be modified to a minor degree (Turecek et al., 2016). Random conjugation of lysine units often leads to a complex mixture of proteins with different number and position of PEG chains, which may also interfere with the receptor/substrate binding (Zaghmi et al., 2019). Although homogenous products can be obtained with purification processes such as chromatography techniques (Pfister and Morbidelli, 2014), a site-specific PEGylation reaction is often preferred.

N-terminal PEGylation is a site-specific reaction based on pKa differences between the ε-amino group of lysine residues (9.3–10.5) and the N-terminal α-amino group of proteins (7.6 to 8) (Dozier and Distefano, 2015). At optimal pH values (generally comprised between 5.5 and 6.5) the N-terminal is unprotonated while lysine residues are predominantly protonated and unable to react (Lee et al., 2003; Gao et al., 2009; Chen et al., 2017). A reductive alkylation with aldehyde derivatives (PEG-aldehyde) proceeds through formation of a Shiff base, and the addition of a reducing agent stabilizes the linkage producing a secondary amine (Hamley, 2014).

Another functional group used for PEGylation is the thiol of cysteine residues. In this case, PEG functionalised with electron-poor olefins (mainly maleimide, but also acrylate, vinyl sulfone) are frequently used to form a thioether bond by Michael-type addition. In order to avoid non-selective coupling with amines, the reaction pH should be carried out at range of 6.5–7.5, values below lysine residues pKa (Dozier and Distefano, 2015; Ravasco et al., 2019). A method related to labeling a disulfide bond between two cysteines was also proposed. The disulfide can be reduced under mild conditions and both the resulting free cysteines react with a bridging PEG-based reagent (Balan et al., 2007; Badescu et al., 2014). Covalent re-bridging of the disulfide bond has the advantage of leaving the protein structurally intact after conjugation.

*O*-glycosylation is a post-translational modification which occurs when a saccharide is covalently bound to a protein through a hydroxyl group of a serine or threonine. *O*-glycosylated proteins can be conjugated to sialic acid- functionalised PEG by sialyltransferase (DeFrees et al., 2006; Dozier and Distefano, 2015). This site-selective modification is therefore obtained at the position that is normally modified with a glycan *in vivo*, and therefore the effect of PEGylation on protein activity is minimized.

### PEGylated Proteins in the Clinic

PEGylated-proteins which have been approved for clinical use or reached the clinical stage are summarized in Table 1. They can be classified as non-specific or site-specific PEGylated proteins.

#### Non-specific PEGylated Proteins

The first PEGylated pharmaceuticals **Adagen**^®^ (pegademase) and **Oncaspar**^®^ (pegaspargase) are actually complex mixtures of various PEGylated species for the treatment of severe combined immunodeficiency, and adequate asparagine depletion in leukemia patients, respectively (Levy et al., 1988; Graham, 2003). In Adagen, the adenosine deaminase was modified with 11–17 molecules of 5 kDa PEG-SS. In Oncaspar, L-asparaginase is covalently conjugated to 69–82 molecules of 5 kDa PEG-SS.

**PegIntron**^®^ is a product based on linear 12-kDa succinimidyl carbonate PEG chains is covalently linked to different sites of Interferon-α 2b (IFN-α2b), via an unstable urethane bond that slowly releases the free protein (Wang et al., 2002; Youngster et al., 2002). In **Pegasys**^®^, a branched 40 kDa PEG-NHS yielded a stable amide bond mainly to four lysine residues of IFN-α2a (Foser et al., 2003).

**Somavert**^®^ (pegvisomant), was approved in 2003 for the treatment of acromegaly (Pradhananga et al., 2002; Parkinson et al., 2003) and it is obtained by nonspecific conjugation of an analog of human growth hormone (hGH) with 4–6 equivalents of PEG-NHS (5kDa). It guarantees an elevated stability to esterase hydrolysis and a half-life approximately 70 h higher than the native protein.

**Mircera**^®^ is an FDA approved (2007) PEGylated erythropoietin with an extended half-life (Macdougall and Eckardt, 2006; Banerjee et al., 2012). It is a mono-PEGylation of a 30-kDa succinimidyl PEG, predominately at lysine or at the N terminus of the protein.

**Krystexxa**^®^ is a hyper-PEGylated product derived by non-human uricase and used to treat gout (Schlesinger et al., 2010; Shannon and Cole, 2012). The conjugation is obtained from PEG *p*-Nitrophenyl carbonate ester, and is necessary to reduce immunogenicity of the non-human enzyme and increase its half-life (Sherman et al., 2008).

**Sylatron**^®^ (peginterferon alfa-2b) was FDA approved in 2011 as adjuvant treatment of melanoma (Herndon et al., 2012; Patel and Walko, 2012) and it is a IFN-α2b conjugate with 12 kDa succinimidyl carbonate PEG (31 kDa).

**Adynovate**^®^ is a PEGylated recombinant factor VIII (rFVIII) approved for hemophilia A and characterized by the prolonged circulatory half-life (Dunn et al., 2018). PEGylation is obtained from lysine residues and optimized to occur in the B-domain which is not required for activity of the protein, thus resulting in an improved pharmacokinetic profile (Konkle et al., 2015).

**Palynziq**^®^ (Pegvaliase) is a phenylalanine ammonia-lyase (rAvPAL) conjugated with linear 20 kDa PEG-NHS. It was recently used in the clinic to treat phenylketonuria, a genetic disorder caused by a lack of phenylalanine hydroxylase causing neurotoxic phenylalanine accumulation (Levy et al., 2018).

PEGylation diminishes immunogenicity and improves pharmacodynamic stability (Longo et al., 2014).

**ADI-PEG 20** is a arginine deiminase (rhArg) conjugate with 10–12 chains of 20 kDa SS-PEG, which has been used against glioblastoma tumor (GBM). Preliminary tests showed that ADI-PEG20 efficiently depleted blood arginine and significantly reduces the growth of GBM in mice, with the advantage that this approach does not require overcoming the blood brain barrier. Although ADI-PEG20 is still under development and not in the market, it is in phase III clinical trials for the treatment of hepatocellular carcinoma, and in phase II studies for acute myeloid leukemia/non-Hodgkin’s lymphoma and for the treatment of metastatic melanoma and some other tumors (Cheng et al., 2007; Tsai et al., 2017).

#### Site-Specific PEGylated Proteins

Filgrastim is an unglycosylated recombinant methionyl human granulocyte colony-stimulating factor (G-CSF), which regulates the production and release of functional neutrophils from the bone marrow.

Two similar products (**Lonquez**^®^ and **Neulasta**^®^) have been recently approved against neutropenia (Piedmonte and Treuheit, 2008; Mahlert et al., 2013). In Lonquez (lipegfilgrastim), the selective addition of PEG in guaranteed through *O*-glycosylation (Mahlert et al., 2013). In Neulasta (pegfilgrastim), methoxy-PEG-propionaldehyde (PEG-aldehyde) is used to obtain selective bioconjugation at the N-terminus via reductive alkylation (Kinstler et al., 2002; Molineux, 2004).

**Cimzia**^®^ (certolizumab pegol) is a PEGylated anti tumor necrosis factor (TNF) recombinant antibody Fab fragment approved for the treatment of rheumatoid arthritis, Crohn’s disease, and axial spondyloarthritis (Blick and Curran, 2007; Nesbitt et al., 2009). The antibody fragment is covalently bound through Michael type addition of PEG2MAL40K moiety which comprises two 20 kDA PEG chains linked to a maleimide group (Chapman et al., 1999). The reactive cysteine is located at three amino acids from the C-terminus of the heavy chain antibody fragment. Due to this site-specific PEG attachment, Cimzia^®^ maintains full binding activity, elevated circulation time and low immunogenicity (Jevševar et al., 2012).

**Plegridy**^®^ is a PEGylated form of IFN β-1a, approved for the treatment of relapsing multiple sclerosis (Chaplin and Gnanapavan, 2015). Glycosylated recombinant IFN β-1a is conjugated with a single linear 20 kDa methoxy PEG-*O*-2-methyl propionaldehyde (44 kDa) moiety at the N-terminus via reductive amination (Baker et al., 2006).

**Refixia**^®^ (nonacog beta pegol), a PEGylated factor IX (rFIX), is used against hemophilia B. The protein is modified by a selective glycoPEGylation (DeFrees et al., 2006; Ezban et al., 2019). Release of the activation peptide by physiologic activators converted the PEGylated recombinant factor IX to recombinant native factor IX and proceeded normal kinetics for factor IX (Østergaard et al., 2011).

**Jivi**^®^ (Damoctocog alfa pegol) and **Esperoct**^®^ (Turoctocog alfa pegol) are site-specific PEGylated (rFVIII) approved for the treatment of hemophilia A (2019; Paik and Deeks, 2019). In Jivi, a single dual-branched 60 kDa PEG molecule is attached to an engineered cysteine residue on the A3 domain of the protein (Castaman and Linari, 2018). The A3 domain was selected to provide a consistent coagulation activity as well as high PEGylation efficiency (Shah et al., 2014). Esperoct is being developed for prophylaxis and treatment of bleeds in hemophilia A patients (Meunier et al., 2017). It is an another B-domain truncated FVIII with a 40 kDa PEG bound by a unique O-linked glycan on the residual 21 amino acid B-domain region (Tiede, 2015; Wynn and Gumuscu, 2016).

### Limits of PEGylation

Despite the widespread clinical use of PEGylated proteins, some important limitations have emerged for clinical applications, which are mainly related to PEG immunogenicity hypersensitivity and non-degradability (Knop et al., 2010; Garay et al., 2012). Here, we critically review the drawbacks associated with pre-existing and induced anti-PEG antibodies, the activation of the complement system and PEG-related cellular vacuolation.

#### PEG Immunogenicity

PEG is generally considered a “stealth” polymer in drug delivery because of its protein-repellent properties, which make conjugated proteins and nanoparticles mostly inert to the biological environment (Yang and Lai, 2015). Steric repulsion and water barrier models are used to explain these characteristics (Zheng et al., 2005). Steric repulsion is mainly attributed to conformational entropy loss due to chain compression as the protein approaches a long PEG chain (McPherson et al., 1998), while water barrier mechanism arises from the large number of water molecules tightly bound (through hydrogen bonds) to the ethylene glycol repeating units, which generate repulsive forces against protein adsorption (Zheng et al., 2004). In these models, chain length, conformation and grafting density are important factors for limiting protein binding (Yang and Lai, 2015). These protein-repellent features should suppress interactions between PEGylated systems and the biological environment, thus PEG conjugation is used to decrease enzymatic degradation, opsonization, and immunogenicity of the protein conjugates.

In contrast to this general assumption, animal studies clearly showed that some PEGylated proteins, particularly ovalbumin and uricase, can elicit antibody formation against PEG (Garay et al., 2012).

In humans, pre-existing and induced antibodies against PEG (anti-PEG) cause an unexpected immunogenic response, also known as the “accelerated blood clearance (ABC) phenomenon (Cheng et al., 1999; Armstrong et al., 2007; Schellekens et al., 2013; Lipsky et al., 2014; Mima et al., 2015). The presence of anti-PEG was correlated with the fast clearance of PEG-asparaginase in patients with acute lymphoblastic leukemia (Armstrong et al., 2007). In a clinical study on the effects of PEG uricase on chronic refractory gout, 40% of patients developed anti-PEG, which was strongly correlated with loss of responsiveness to this protein conjugate (Lipsky et al., 2014).

In a recent study, pre-existing anti-PEG was identified in over 25% of healthy blood donors (Armstrong, 2009), in contrasts with only 0.2% occurrence reported over 20 years ago by Richter and Åkerblom (1984). This increase may be explained as a result of the large amount of PEG that is present nowadays in cosmetics, pharmaceuticals and processed foods. The continuous exposure to these products may induce anti-PEG antibodies in humans (Garay et al., 2012), although the constant analytical improvements of antibody detection over the years may also explain some discrepancies among different tests.

Different studies have shown that pre-existing and induced anti-PEG may bind to the PEG backbone (Richter and Åkerblom, 1984; Armstrong, 2009). However, since PEGylated therapeutic proteins generally contain methoxy-terminated PEG (mPEG), it has been hypothesized that antibodies with high affinity for methoxy groups may also be involved (Garay et al., 2012). Using hydroxy-PEG (HO-PEG) instead of mPEG in preparing conjugates of albumin, human interferon-α, and porcine uricase, a reduced immunogenicity was found in rabbits (Sherman et al., 2012). On the other hand, *in vitro* studies demonstrated that OH-PEG is a stronger complement activator than mPEG, since the hydroxyl group is able to covalently bind to the complement component C3 (Reddy et al., 2007). PEG-induced complement activation requires further investigation. Anti-PEG binding can trigger opsonization of complement factors, which subsequently promote phagocytosis by the mononuclear phagocyte system (Verhoef et al., 2014). Moreover, other studies on PEGylated therapeutics reported non-antibody-mediated complement activation, either by the mannose-binding lectin pathway or the alternative pathway (Verhoef et al., 2014).

Further studies are therefore required to determine the specificity of anti-PEGs, how these antibodies can influence the pharmacokinetics of PEGylated proteins, and how the complement activation by the polymer may cause severe hypersensitivity reactions.

#### Safety of PEGylation

The molecular weight of the conjugated PEG is typically selected to avoid renal clearance, and therefore to obtain an elevated half-life of the therapeutic proteins (Verhoef et al., 2014). However, the non-degradability of PEG in systemic circulation may lead to polymer accumulation *in vivo*. After repeated administration of some approved PEGylated biopharmaceuticals, cellular vacuolation were histologically observed in certain organs and tissues (Ivens et al., 2015). Vacuolation is considered a normal physiological process by which various cell types attempt to remove foreign materials (Stidl et al., 2018). In mammalian cells, vacuoles are formed in different cellular compartments (e.g., endosomes, lysosomes, endothelial reticulum), and this phenomenon can be transient or irreversible (Stidl et al., 2018).

PEG-associated vacuolization in macrophages, predominantly within the reticuloendothelial system, is well documented with no detectable toxicological relevance (Kronenberg et al., 2013). However, several preclinical toxicology studies on approved PEGylated therapeutics provided evidence of vacuolation in renal tubule cells and epithelial cells of the choroid plexus (Stidl et al., 2016; Stidl et al., 2018). In one study, high doses of tumor necrosis factor binding protein (TNF-bp) conjugated with a 20 kDa PEG caused vacuolation of renal cortical tubular epithelium cells in rats, over a period of 3 months (Bendele et al., 1998). Tubular vacuolation caused distortion of tubular profiles and compression of nuclei, without leading to necrosis (Bendele et al., 1998; Stidl et al., 2016). Renal tubular cell vacuoles and splenic vacuolated macrophages were also reported for hemoglobin (Hb) conjugated to a 5 kDa PEG administered in rats (Conover et al., 1996). A serious concern is the vacuolation in the epithelial cells of the choroid plexus, which is the main source of cerebrospinal fluid and a key component of the blood-cerebrospinal fluid barrier (Stidl et al., 2018). Recently, a correlation between the molecular weight of unconjugated linear PEG (from 10 to 40 kDa) and vacuolation in rats was reported after repeated injections for 3 months (Rudmann et al., 2013). It was observed that the highest molecular weight PEG (40 kDa) triggered vacuolation in macrophages, choroid plexus epithelial cells and renal tubular epithelial cells. Immune-historeactivity to PEG decreased in renal tubule cells, but increased in splenic macrophages and choroid plexus epithelial cells (Rudmann et al., 2013).

Due to the diversity of marketed PEGylated proteins and new conjugates under development, nonclinical toxicology studies are therefore important to determine tissue location, reversibility, and severity of vacuolation with its possible functional consequences, in order to evaluate potential patient safety risks (Ivens et al., 2015).

### Non-degradable PEG Alternatives

Although PEGylated proteins are the only protein-polymer conjugates approved for clinical use, many other biocompatible polymers have been recently investigated as an alternative to PEG, which showed promising results *in vitro* and *in vivo*.

**Poly(vinyl pyrrolidone) (PVP)** and **Poly(N-(2-hydroxypropyl) methacrylamide) (PHPMA)** are non-biodegradable, nonionic and non-immunogenic polymers, well-established as biocompatible drug carriers. They have been recently synthesized via Reversible Addition Fragmentation Chain Transfer (RAFT) to obtain narrow molecular weight distributions, which are ideal for bioconjugation (Scales et al., 2005; Zelikin et al., 2007). PVP- conjugated TNF-α provided longer circulation than PEG-TNF-α at the same molecular weight (Kaneda et al., 2004). HPMA copolymer–insulin and HPMA copolymer–chymotrypsin conjugates were also investigated (Kopecek and Kopecková, 2010).

**Polyglycerol (PG)** showed similar characteristics to PEG in terms of non-degradability, protein repellence, and superior biocompatibility and toxicity profile (Kainthan and Brooks, 2007; Imran ul-haq et al., 2012). Linear and hyperbranched PG were conjugated to model proteins (bovine serum albumin (BSA) and lysozyme) to assess their effect on conjugate activity (Wurm et al., 2012).

**Polyoxazolines (POZs)** are biocompatible polymers with ‘stealth’ properties and easy renal clearance (Zalipsky et al., 1996; Gaertner et al., 2007). Bioconjugation between poly(2-ethyl-2-oxazoline) and G-CSF, a hemopoietic cytokine, through reductive amination or enzyme-mediated acyl transfer, resulted in bioactive conjugates *in vivo* (Mero et al., 2012). POZs with methyl, ethyl and propyl side chains were synthesized by living cationic polymerisation and conjugated to BSA and insulin (Viegas et al., 2011) obtaining low immunogenicity and longer blood glucose control than native insulin in rats.

**Poly(*N*-acryloylmorpholine) (PNAM)** is a biocompatible water-soluble acrylamide derivative which can be synthesized via RAFT polymerisation and modified for attachment to enzymes in order to reduce immunogenicity. Monovalent lysozyme-PNAM conjugates with relatively low molar mass polymers displayed equal or even higher activity than the native protein, while all conjugates showed an improved protein solubility (Morgenstern et al., 2018).

### Degradable PEG Alternatives

**Polysialic acid (PSA)**, also known as columinic acid, is a linear small polysaccharide containing α-2,8-linked sialic acid (neurominic acid) with (*n* = 8 to >100) residues. PSA-conjugated L-asparaginase, obtained by reductive amination, reduced the antigenicity of asparaginase and prolongs the circulation half-life in mice (Fernandes and Gregoriadis, 2001). PSA conjugated to insulin on the N-terminus and lysine residues improved pharmacological properties and provided a more accurate long-term control of blood glucose levels (Jain et al., 2003).

**Trehalose glycopolymers** enhance *in vivo* plasma half-life and enhance stability on storage. Insulin-trehalose glycopolymer conjugate showed similar insulin-PEG prolonged plasma circulation in mice and low toxic effects (Liu et al., 2017; Mansfield and Maynard, 2018).

Biodegradable polysaccharides, such as **alginate** (Mondal et al., 2006) and **hyaluronic acid (HA)** (Mero and Campisi, 2014), have been explored for protein conjugation. As for SS-PEG, random lysines conjugation showed critical purification, reproducibility drawbacks, and lost in activity (Ferguson et al., 2010). The partial periodate oxidation of some saccharide repeating units generates aldehyde groups which allows selective N-terminal reductive amination. This approach was used to selectively modify insulin, hGH and INFα (Yang et al., 2011, 2012). A site selective conjugation of insulin and IFNα was also obtained by introducing an aldehyde group in the polymer backbone without altering the HA integrity (Mero and Campisi, 2014). In diabetic rats, HA-insulin conjugates maintained a glucose lowering effect up to 6 h, while free insulin was inactive after 1 h. Unexpectedly, when an elevated amount of insulin was conjugated, its effect on blood glucose level decreased, probably because of a steric entanglement affecting the receptor/protein recognition (Mero and Campisi, 2014).

**Hydroxyethyl starch (HES)** is a biodegradable FDA approved polymer, whose non-immunogenicity is possibly attributed to structural similarities with glycogen (Paleos et al., 2017). HES is degraded by α-amylase in the plasma, which can be controlled by modifying the molar mass and the degree of hydroxyethylation. Its conjugates have been extensively investigated for therapeutic uses (Ko and Maynard, 2018). The HESylation of erythropoietin (EPO) had comparable *in vitro* and *in vivo* activities to PEGylated-EPO (Mircera) (Hey et al., 2012; Pelegri-O’Day et al., 2014). The conjugation of HES to G-CSF and INF-α have also shown comparable results (Hey et al., 2012). Furthermore HESylation^®^ sharply improved the storage stability over PEGylation by remaining totally amorphous during lyophilisation, with and without lyoprotectants (Liebner et al., 2015).

Protein conjugation with biodegradable **poly(ethyl ethylene phosphate) (PEEP)** was also reported (Steinbach et al., 2017). PPEylated BSA and catalase showed comparable activity to their PEG-equivalent.

**Recombinant synthetic polypeptides**, are biomimetic polymers with tunable degradability, versatile side chain functionalities, and self-assembly behaviors. They can be conjugated with proteins either by chemical coupling or by genetic engineering approach. The hGH fused with the synthetic polypeptide XTEN^TM^ (Schellenberger et al., 2009) (hGH-XTEN) is undergoing a Phase II clinical trial as monthly administration for the treatment of hGH deficiency. Elastin-like polypeptide (ELP) fused with IFN-α was able to prolong the circulating half-life of the protein (Hu et al., 2015). A randomized sequence of proline, alanine and serine (PAS) guaranteed properties remarkably similar to PEG when they were fused to therapeutic proteins, including GF, hGH, Leptin (Schlapschy et al., 2013; Gebauer and Skerra, 2018). PASylated-hGH exhibited 94-fold longer plasma half-life in mice than the native protein (Gebauer and Skerra, 2018), and it led to a 2.8-fold higher IGF-1 plasma concentration compared with the mice treated with hGH (Schlapschy et al., 2013). Kidney, liver, and spleen showed no histological changes after the treatment, and repeated dose administration confirmed the absence of immune reactivity toward the PAS moiety (Schlapschy et al., 2013). Artificial gelatin-like peptidic sequence (GLK) was fused to granulocyte-colony-stimulating factor (G-CSF) in order to generate a chimeric GLK/G-CSF fusion protein with enhanced plasma half-life (Huang et al., 2010). The polypeptoid Polysarcosine (PSar) has been recently considered an emerging “stealth” biodegradable polymer for many biomedical applications (Chan et al., 2018). A N-terminal specific polysarcosine-interferon conjugate (PSar-IFN) showed significantly more potency in inhibiting tumor growth, and elicited considerably less anti-IFN antibodies in mouse than its PEGylated counterpart (Hu et al., 2018).

### Grafting Methods

**FIGURE 2:**
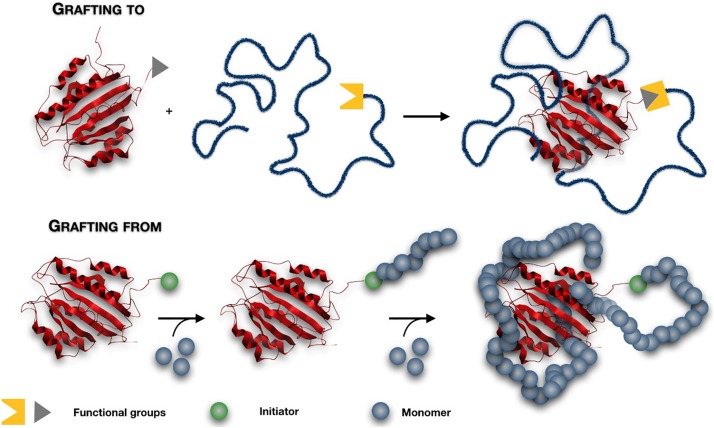
The ‘grafting to’ and ‘grafting from’ conjugation strategies. In a ‘grafting to’ method, end-functionalised polymers are firstly synthesized and then attached to the protein via a conjugation reaction. In a ‘grafting from’ method, a low molecular weight initiator is firstly attached to the protein and then the polymerization is initiated directly from the protein.

In all the materials discussed above, end-functionalised polymers are firstly synthesized and then attached to the protein via a conjugation reaction. This strategy is generally called ‘grafting to,’ and it is generally characterized by low conversion, due to the steric hindrance and the low concentration of the reactive groups (Francis et al., 1998). An excess of reactive polymer is generally needed, therefore an efficient purification step to remove the unbound polymer is required (Wallat et al., 2014). Recently, an alternative ‘grafting from’ approach has been proposed to overcome these drawbacks. This method consists of initiating the polymerization directly from the surface of proteins, obtaining finely controlled products (Magnusson et al., 2010) (Figure 2). A low molecular weight initiator is firstly attached to the protein via bioconjugation. Due to the small size of this molecule, the steric hindrance that occurred between two ‘giant’ macromolecules during the “grafting to” method is avoided, and an excellent yield of protein-polymer conjugates can be obtained (Salmaso and Caliceti, 2011). The purification process of high molecular weight conjugates from the unreacted small molecular monomers and catalyst is easier and faster (Pelegri-O’Day and Maynard, 2016; Kovaliov et al., 2018).

Controlled-living polymerisation techniques such as Atom transfer radical polymerization (ATRP) and RAFT have been recently explored for site-specific polymer growth in aqueous solvent, ambient temperature, and physological pH, i.e., conditions that are well tolerated by biomolecules (Averick et al., 2011). The main drawbacks are related to the challenges in controlling the polymerisation process under bio-relevant conditions. Activator Generated by Electron Transfer (AGET) ATRP has been recently developed to synthesize polymer-protein conjugates through polymerization of PEG methacrylate (PEGMA) macromonomers, from initiator-functionalized recombinant hGH (Magnusson et al., 2010) and trypsin (Yaşayan et al., 2011). Activator ReGenerated by Electron Transfer (ARGET) ATRP in aqueous media, has shown promising results for conjugation of therapeutic proteins (Simakova et al., 2012) achieving narrow molecular weight distributions (Mw/Mn < 1.3).

A PEG-based polymer grafted from the C-terminus of INFα, obtained by ATRP of poly(oligo(ethylene glycol) methyl ether methacrylate) (POEGMA), was used to treat a murine cancer model. The POEGMA-INFα conjugate completely inhibited and eradicated tumors of 75% mice without appreciable systemic toxicity, whereas at the same dose, no mice treated with the PEGASYS^®^ survived for over 58 days (Hu et al., 2016).

TL lipase was modified with ATRP initiators either at the amine side chain of lysine or acid residues of aspartic and glutamic amino acids, and *N*-[3-(*N,N*-dimethylamino)propyl] acrylamide (DMAPA) was grafted-from by Continuous Activator Regeneration (ICAR) ATRP (Kovaliov et al., 2018). The activity was higher for both conjugates compare to native protein.

Alternatively, photoinduced electron transfer reversible addition-fragmentation chain transfer (PET-RAFT) polymerisation of DMAPA was successfully used on TL lipase without affecting its activity (Kovaliov et al., 2018). RAFT polymerisation allowed to obtain well-defined poly(*N*-isopropylacrylamide) linked with BSA (Li et al., 2011a) and lysozyme–poly(*N*-isopropylacrylamide)-*b*-poly(*N,N*-dimethylacrylamide) block copolymer conjugates (Li et al., 2011b).

## Liposomes

Liposomes are phospholipid vesicles which consist of at least one lipid bilayer enclosing a discrete aqueous domain. While hydrophobic compounds can be inserted into the lipid membrane, hydrophilic molecules can be entrapped in the aqueous core, and this characteristic enables low and high molecular weight biomolecules to be encapsulated and later released at the targeted site (Sercombe et al., 2015; Huang et al., 2017). Liposomes represent the first nanosized drug delivery system which made the transition from bench to clinical application, and provide ideal characteristics of biocompatibility, biodegradability, variable compositions (Allen and Cullis, 2013; Sercombe et al., 2015). Among their advantages, liposomal formulations can be administered through several different routes such as parenteral (the most studied), oral (He et al., 2019), pulmonary (Khanna et al., 1997b), nasal (Luo et al., 2018), ocular (Agarwal et al., 2016), and topical (Yarosh et al., 2001).

**FIGURE 3:**
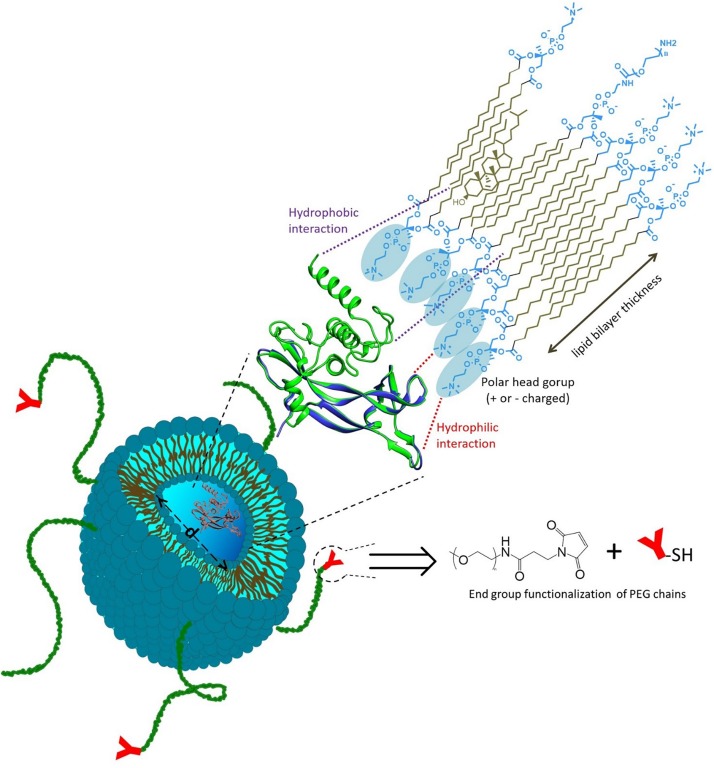
Liposome designed for therapeutic protein delivery. Protein is generally entrapped within the liposome core (of tunable diameter d), and its encapsulation may also involve hydrophilic/hydrophobic interactions with the lipid bilayer. Liposomes can be PEGylated to prolong circulation *in vivo*, and may be conjugated with active ligands to provide active targeting.

Liposome surfaces can be easily functionalised with an appropriate ligand for targeted delivery and also decorated with protein-repellent polymers, such as PEG, to inhibit opsonization and clearance by the mononuclear phagocytic system (Immordino et al., 2006; Hwang et al., 2012; Pattni et al., 2015). Due to the fast development of nanomedicine, several protein delivery formulations based on liposomes have been developed for therapeutic use. Once entrapped in liposomes, a therapeutic protein may increase its stability, as the lipid bilayer provides protection from degradation (Figure 3) (Tan et al., 2010). Liposomes can be PEGylated to prolong circulation *in vivo*, and may be conjugated with active ligands to provide active targeting (Hatakeyama et al., 2013). Some protein-loaded liposomes reached the clinical trials and some products are already on the market. However, compared with protein-polymer conjugates, a limited quantity of protein-loaded liposomes has been approved for marketing, and the majority of current liposomal protein formulations are still in preclinical stages (Table 2). In fact, although liposomes are good candidates for *in vivo* delivery of high molecular weight compounds (such as protein/peptide drugs and nucleic acids), their nanoencapsulation is often hindered by instability issues during the liposome preparative process and storage, as well as by the low encapsulation efficiencies (Xu et al., 2012; Huang et al., 2017), as discussed hereafter.

**TABLE 2:** List of protein-loaded liposomes in clinical use.

**Commercial name**	**Active protein**	**Treatment**	**Company**	**Status**	**Year**	**References**
Epaxal	Hepatitis A virus proteins	Hepatitis-A	Crucell (former Berna Biotech Ltd.)	On market	1994	Cryz, 1999
Inflexal-V	Influenza virus proteins	Trivalent influenza vaccine	Crucell (former Berna Biotech Ltd.)	On market	1997	Herzog et al., 2009
Curosurf	SP-B and SP-C proteins	Lung activator for stress disorder	Chiesi Farmaceutici	On market	1999	Walther et al., 2000
T4N5 liposome lotion	T4 endonuclease V (T4N5) enzyme	Skin cancer	AGI Dermatics Inc.	Phase III	2007	Bulbake et al., 2017
Hepatic-directed vesicles-insulin (HDV-I)	Insulin	Diabetes	Diasome Pharmaceuticals	Phase II	2019	Diasome_Pharmaceuticals, 2019
Biphasix	INF-α	Genital warts and cervical dysplasia	Altum Pharmaceuticals	Phase I/II	2011	Altum_Pharmaceuticals_Inc, 2019
IL-2 liposomes	IL-2	Pulmonary metastases	Biomira United States	Phase I	2000	Skubitz and Anderson, 2000

### Protein-Loaded Liposomes in the Clinic

The first protein-liposome systems accepted for clinical use were virosomes, i.e., drug/vaccine delivery systems based on unilamellar phospholipid membrane which incorporate virus-derived proteins. **Epaxal**^®^ was the first commercially available liposomal vaccine, which consists of particles of ∼150 nm composed of phosphatidylcholine and phosphatidylethanolamine lipids, neuraminidase, hemagglutinin and inactivated hepatitis A virus. The hemagglutinin and the neuraminidase bind strongly to the lipid layer by a non-covalent bond, stabilize the liposomal structure, and target the liposome to immune-competent cells (Cryz, 1999; Bovier, 2008). Epaxal has demonstrated safety and efficacy in clinical studies, and is licensed in several countries. **Inflexal-V**^®^ is present on the market since 1997 in many countries (with different commercial names) as a therapy against flu. It is similar to Epaxal as it consists of unilamellar bilayer liposomes of about 150 nm, made of phosphatidylcholine, and the mixture of three monovalent virosome pools, each formed with one influenza strain- specific hemagglutinin and neuraminidase glycoproteins (Herzog et al., 2009).

**Curosurf**^®^ (poractant alfa) is a product (FDA approved in the ’90s) composed by sterile suspension for endotracheobronchial instillation of animal-derived lipids used for the treatment of neonatal respiratory distress syndrome. This product is composed of phosphatidylcholine, dipalmitoylphosphatidylcholine and the small hydrophobic surfactant proteins SP-B (8.7 kDa) and SP-C (3.7 kDa). Through a reorganization of the lipids present in the fluid that covers the lung, the alveoli can swell more easily, thus preventing the alveolar collapse. The two proteins are essential to reduce the surface tension at the air-water interface by the formation of a surface-active film highly enriched in dipalmitoylphosphatidylcholine (Walther et al., 2000). The particle size is variable and different studies reported values between 35 μm to 50 nm (uni- and multilamellar vesicles) (Waisman et al., 2007).

**T4N5 liposome lotion** (Dimericine) is based on T4 endonuclease V enzyme loaded into egg lecithin liposomes. The T4 endonuclease V enzyme repairs the damaged DNA preventing the first stage of skin cancer (Bulbake et al., 2017; Jeter et al., 2019). Phase I/II trials indicated effective prevention of skin cancer in Xeroderma pigmentosum patients. However, phase III trials were terminated in 2009 with lack of expected clinical outcomes (Bulbake et al., 2017).

**Hepatic-directed vesicles-insulin (HDV-1)** is a liposomal delivery system for diabetes treatment via oral and subcutaneous routes, which have been tested in phase II clinical trials (Diasome_Pharmaceuticals, 2019). These insulin-loaded liposomes (size < 150 nm), contain the proprietary hepatocyte-targeting molecule (HTM) and biotin-phosphatidylethanolamine lipids. In diabetic animal models, it was an effective insulin-replacement treatment as it showed very low toxicity and successfully targeted the hepatocytes in the liver (Geho et al., 2009).

**Biphasix**^TM^ is a topical formulation that is intended to be easily self-applied to human papillomavirus (HPV) -infected tissues, to deliver IFN-α into the skin and mucosal tissues. It regards with the encapsulation of the therapeutic protein in multilayered, lipid-based submicronvesicles (Altum_Pharmaceuticals_Inc.; Roohnikan et al., 2019). These vesicles have complex structures that include a variety of compartments into which drug molecules can be integrated, and the emulsion is completed with other excipients typical of a topic formulation. It has completed phase I and II clinical trials, where it was shown to be active (in cervical neoplasia regression) with no systemic or local side effects (Altum_Pharmaceuticals_Inc.).

**IL-2 liposomes** are interleukin-2 loaded liposomes which have been tested in phase I clinical trials (Skubitz and Anderson, 2000). This liposome preparation contains a synthetic lipid, dimyristoylphosphatidyl choline (DMPC), human serum albumin and human recombinant IL-2 (Khanna et al., 1997a). Administration by inhalation showed a significant increase in bronchoalveolar lavage leukocytes in the lung compared to free IL-2 administered via conventional routes due to a high concentration of the drug at the specific site of action (Khanna et al., 1997b).

### Liposome Composition for Protein Delivery

In general, the liposome composition includes lipids of natural origin (e.g., egg lecithin, cholesterol), synthetic, or semi-synthetic [e.g., lipids manufactured by modification of naturally occurring precursors such as dipalmitoylphosphatidylcholine (DPPC), distearoylphosphatidylcholine (DSPC), or dimyristoyl- phosphatidylcholine (DMPC)] (Olusanya et al., 2018).

Conventional liposomes have a short circulation time *in vivo*, since they are quickly uptaken and eliminated by mononuclear phagocyte system. PEGylation is also used in liposomes to inhibit the opsonization, thus extending blood-circulation. This effect can be modulated by the molecular weight of the PEG chains and the grafting density at the liposome surface (Wang et al., 2016).

PEGylated (stealth) liposomal formulations have been studied for protein delivery, for instance as safe and effective means to deliver protein antigens (tetanus toxoid (TT), ovalbumin) to potent antigen-presenting dendritic cells for the induction of CD4+ and CD8+ T-cell response *in vivo* (Ignatius et al., 2000). Hemoglobin (LEH)-loaded liposomes, prepared with anionic lipid hexadecylcarbamoylmethyl-hexadecanoate (HDAS), cholesterol and HDAS-conjugated PEG2000, were tested as oxygen nanocarriers, and succeed in preventing systemic inflammation and multi-organ injuries caused by hemorrhagic shock in mice (Yadav et al., 2016).

Similarly to PEG-protein conjugates, PEGylation also presents undesirable effects in liposomes. For example, PEG steric effects reduce the interaction of liposomes with the cell membrane or tissue extracellular matrix when specific targeting is required (Hatakeyama et al., 2013). Ligands such as antibodies, protein fragments, peptides and aptamers are often conjugated to the terminal group of the PEG chains which are attached to the liposome surface, in order to respond to the extracellular or intracellular environment, thus obtaining active targeting (Hatakeyama et al., 2013; Fang et al., 2017). Several papers have been dedicated to PEG-ligand conjugation of liposomes for the release of low molecular weight drugs (Eloy et al., 2014; Noble et al., 2014; Belfiore et al., 2018) and this approach has also been used for protein nanoencapsulation (e.g., trypsin and chymotrypsin inhibitor into PEGylated liposomes conjugated with transferrin) (Joanitti et al., 2018). However, it is worth noting that functionalization of liposomes with various targeting ligands has resulted in enhanced detection by the immune-system, and that targeting capability may be compromised by the interaction between serum-protein and ligands (Riaz et al., 2018).

### Methods for Preparing Liposomes

**FIGURE 4:**
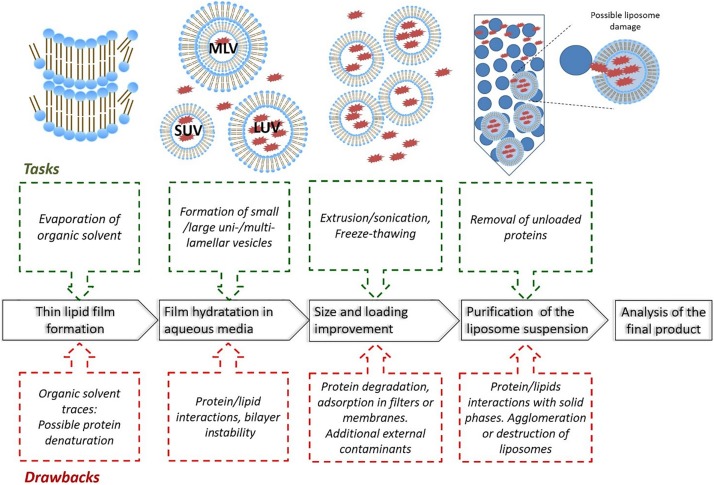
Main steps involved with the preparation of protein-loaded liposomes, including typical tasks and drawbacks (MLV: Multilamellar Vesicles, LUV: Large unilamellar vesicles, SUV: small unilamellar vesicles).

Different methods of liposomes preparation have been reported to optimize the drug encapsulation and to obtain a homogenous particle population, such as mechanical dispersion methods, solvent dispersion methods, and detergent removal methods (Vemuri and Rhodes, 1995; Akbarzadeh et al., 2013). The poor protein stability during preparation, especially when organic solvents and detergents are used, generally limit the preparative choice to the mechanical dispersion methods (Xu et al., 2012). In most cases, the procedure to prepare protein-loaded liposomes is based on the following steps (as summarized in Figure 4): firstly, a thin lipid film is formed or dried from organic solvents, then the film/solid is hydrated with dispersed-protein aqueous media. In this step, liposomes of different sizes and/or uni-, bi- and multi-lamellar vesicles are obtained. A further step is dedicated to the size homogenisation (mainly by extrusion or sonication) and improvement of drug loading (typically by freeze-thawing), then the liposomes are purified and characterized (Xu et al., 2012; Akbarzadeh et al., 2013).

Compared with low molecular weight drugs, the encapsulation of large biomolecules such as proteins and peptides generally lead to several drawbacks, such as low encapsulation efficiency, irregular particle size distributions, the presence of organic solvent residues or metal ions, which can affect the protein stability and the safety of the clinical treatment. The purification also represents a critical step; size exclusion or dialysis are the most used methods, and possible liposome interaction with the stationary phases or membranes should not be excluded. When centrifugation is used, the right choice of the centrifugal speed is necessary to avoid the formation of agglomerates or liposome destruction. The sterilization of liposomal preparations is also a critical issue (Meyer et al., 1994; Heeremans et al., 1995), as well as storage conditions. Liposome suspensions should be stored in a refrigerator, as a freezer will lead to formation of ice crystals that may rupture the phospholipid membrane (Riaz et al., 2018). Different preparative methods have been reported in literature for *in vivo* applications, with results showing significant differences in terms of size distribution and encapsulation efficiency (Table 3).

**TABLE 3:** Lipid formulations, preparative methods and characterisation of protein-loaded liposomes for *in vivo* applications.

**Protein**	Formulation*	**Method**	**Size <d> [nm]**	**Encap. Efficiency (%)**	***In vivo* study**	**References**
rhG-CSF	DMPG:DSPC:Chol	Lipid film hydration/microfluidisation/centrifugation Lipid powder hydration/microfluidisation/dialysis Lipid film hydration/sonication/freeze-thawing/dialysis	250 340 760–780	2 30 80–90	Subcutaneous injections in rats	Meyer et al., 1994
OVA and TT	Chol:POPC:PE-PEG2k	Thin film hydration and extrusion	∼100	–	Immunization of mice	Ignatius et al., 2000
bFGF	PC/Chol	pH gradient method Ammonium sulfate gradient method Reverse-phase evaporation method Thin film method	∼120	81.6 65.7 69.5 58.6	Wound healing in rats	Xiang et al., 2011
NGF	PC/Chol DSPE-PEG2k-RMP-7/DSPE-PEG2k	Reverse phase evaporation	64–73	24–34	Transport across BBB in rats	Xie et al., 2005
(FTIC-) BSA	PC/Chol/DSPE-PEG2k/S-PEG-polySDM/Rh-DHPE	Thin layer rehydration and extrusion	167–287	18–20	Bladder epithelium targeting in mice	Vila-Caballer et al., 2016
BSA	PC:Chol:DSPE-PEG.	Thin film hydration, Freezing-thawing and extrusion	208–346	41–45	Safety and pharmacokinetic studies in mice	Okamoto et al., 2018
hIgG	PC/Chol	Dehydration-rehydration	219–230	30–31	Biodistribution in mice	García-Santana et al., 2006
Hb	HDAS:Chol:HDAS-PEG2k	High pressure homogenization method	216	<5	Hemorrhagic shock in rats	Yadav et al., 2016

* 1,2-dimyristoyl sn-glycero-3-phosphocholine (DMPG); 1,2-Distearoyl-sn-glycero-3-phosphocholine (DSPC); cholesterol (Chol); 1-Palmitoyl-2-oleoyl-sn-glycero-3-phosphocholine (POPC); 1,2-dipalmitoyl-sn-glycero-3-phosphoethanolamine-*N*-[methoxy(polyethylene glycol)-2000] (PE-PEG2k), L-α-phosphatidylcholine (PC); RMP-7-conjugated- 1,2-dioleoyl-sn-glycero-3-phosphoethanolamine-n-[poly(ethyleneglycol)]-hydroxy succinamide (DSPE-PEG2k-RMP-7); 1,2-distearoyl-sn-glycero-3-phosphoethanol-amine-*N*-[methoxy-poly(ethyleneglycol) 2000], (DSPE-PEG2k); stearoyl-poly(ethylene glycol)-poly(methacryloyl sulfadimethoxine) copolymer, (S-PEG-polySDM); *N*-(Lissamine Rhodamine B sulfonyl)-1, 2-dihexadecanoyl-sn-glycero-3-phosphoethanolamine triethylammonium salt, (rh-DHPE); hexadecylcarbamoylmethylhexadecanoate, (HDAS); Hexadecylcarbamoylmethylhexadecanoate-PEG2000 conjugated, (HDAS-PEG2k).

The encapsulation of granulocyte colony-stimulating factor (rhG-CSF) was obtained by using three different preparative methods (lipid film hydration- microfluidisation- centrifugation, lipid powder hydration- microfluidisation- dialysis, and lipid film hydration- sonication- freeze-thawing- dialysis). Results showed that encapsulation efficiency increased with the size of the nanocarriers, and that these liposomes were successful in releasing rhG-CSF in rats (Meyer et al., 1994). In this work, a rapid protein release (100% within 24 h) or a much slower release (50% in 4 days) was obtained *in vivo* by varying the lipid composition (DPPC or DSPC:cholesterol, respectively).

The encapsulation of ovalbumin (OVA), tetanus toxoid (TT), bovine serum albumin (BSA), glutathione S-transferase (GST), human gamma-globulin (hIgG) by different techniques also showed marked differences in size distribution and encapsulation efficiency (Ignatius et al., 2000; García-Santana et al., 2006; Ahn et al., 2009; Vila-Caballer et al., 2016; Okamoto et al., 2018; Forbes et al., 2019; Hussain et al., 2019). More specific therapeutic proteins have been encapsulated in liposomal formulations to improve release at a specific site. Basic fibroblast growth factor (bFGF), nerve growth factor (NGF), hemoglobin (Hb) are some of the biomolecules examined (Xie et al., 2005; Xiang et al., 2011; Yadav et al., 2016). It was observed that by using either a pH gradient method or freeze-thawing followed by extrusion, similar bFGF encapsulation yield (∼80%) were obtained (Xiang et al., 2011).

Nowadays, new methods have emerged with the aim of improving the encapsulation degree without affecting the integrity of the biomacromolecules. The use of supercritical carbon dioxide (ScCO_2_) as a non-toxic substitute for organic solvents have led to some potential applications in the pharmaceutical industry for the micro- and nano-encapsulation of drugs (Santo et al., 2014; Trucillo et al., 2019). The encapsulation in liposomes of several payloads including antibodies and albumin was obtained using a ScCO_2_-assisted process (Santo et al., 2014) with high encapsulation efficiency (>90%) (Trucillo et al., 2019).

The microfluidic-based system is a promising method to prepare protein-loaded liposomes for a rapid and scale-independent manufacture, which incorporated in-line purification and particle size monitoring. A range of neutral and anionic protein-loaded liposomes was obtained with protein efficiency (20–35%) higher than conventional methods (sonication or extrusion, <5%) and presented smaller and homogenous particle size between 60 and 100 nm (Forbes et al., 2019).

### Protein Encapsulation Efficiency

The low encapsulation efficiency in small-sized liposomes represents a major challenge in the development of liposomal drug delivery systems for therapeutic proteins (Xu et al., 2012). The nanoencapsulation of large macromolecules is predominately limited by the low entrapment volume (which depends on particle size), however, other factors are also crucial for the protein encapsulation efficiency, such as lipid composition and molar ratio, concentrations, buffer solution pH and ionic strength, preparative method, as well as the protein nature, its hydrodynamic diameter and concentration. In studies carried out with phosphatidylcholine and tissue-type Plasminogen activator (t-PA), higher encapsulation yields were obtained at higher lipids concentration, lower ionic strength larger liposome size (Heeremans et al., 1995). The effects of lipid composition concentration, buffer pH, ionic strength, protein size, liposome size and surface charge were evaluated on trypsin, horseradish peroxidase, enterokinase and hyaluronidase as model enzymes with different molecular weights and isoelectric points (Hwang et al., 2012). Results confirmed the behavior reported by Heeremans on the effect of lipid concentration and the particle size, and also showed that the encapsulation yield did not depend of the protein molecular weight, it was relatively low in any case (approximately 5–20%), and that basic pH and lower ionic strength favored the encapsulation of all proteins (Hwang et al., 2012).

The effect of protein interactions with the lipid membrane on the encapsulation efficiency is still a point of discussion among scientists. In fact, the protein may be surrounded by the lipid membrane or occupy the hydrophobic transmembrane region, depending on the nature of the proteins and the lipids involved, which are capable of forming electrostatic and hydrophobic interactions, hydrogen bonds, due to the polar and hydrophobic groups present in their complex structure (Lee, 2004; McClements, 2018). Computational simulations have also been used for a deeper understanding of protein-lipid interaction (Khan et al., 2016; van‘t Hag et al., 2016).

### Stimuli-Responsive Liposomes

In liposomes, the release of proteins is generally controlled by physicochemical mechanisms such as lipid dissociations and simple diffusion (Lu et al., 2014). Recently, stimulus-responsive liposomes have been studied for the release of conventional drugs, and more recently for large biomolecules such as proteins and peptides. Different activation methods (temperature, pH, enzyme, redox, and light) have been used to confer stimuli-responsive properties. pH-responsive liposomes can be used for targeted release when the pathological site presents altered pH compared with normal tissues. The slightly pH change can trigger deformations in the permeability of the liposomal membrane due to the presence of pH-sensitive moieties which produce morphological changes of the lipid bilayers and consequent release of the payload. Lipids such as oleic and hyaluronic acid, derivatives of succinic acid, and other pH-sensitive phospholipids can be used for the release of therapeutic proteins, for the release in solid tumors or in the bladder cavity (Vila-Caballer et al., 2016).

Thermosensitive liposomes (TSL) are another example of “smart” nanocarriers as temperature changes can be used as “trigger” at the diseased site. Such liposomes are composed of phospholipids that present a gel-to-liquid crystalline phase transition temperature (Tm) slightly above the physiological temperature. When mild hyperthermia (a local increase of temperature up to 42°C) is applied, the lipid bilayer will ‘melt’ to a fluid state upon arrival in the heated targeted area, and in that process liposomes rapidly release their payloads (Al-Ahmady and Kostarelos, 2016). Several lipids present a low-temperature transition, DPPC is the most common thermosensitive lipid which presents a Tm close to 41°C (Mazzotta et al., 2018). DSPE-PEG2000 also helped to stabilize the lipid membrane at physiological temperature and to enhance the kinetics release at 40–41°C (from 10 to 40% after 2 h incubation) (Huang et al., 2017). Listeriolysin *O*-loaded thermosensitive immunoliposomes were developed to release the payload when heated slightly above body temperature (Kullberg et al., 2005). Small unilamellar LTSL loaded with mistletoe lectin-1 (ML1), a ribosome-inactivating protein with potent cytotoxic activity in tumor cells, showed protein release (15–46%) after a 15-min heating period at 41–42°C (de Matos et al., 2018).

## Alternative Nanocarriers for Protein Delivery

**FIGURE 5:**
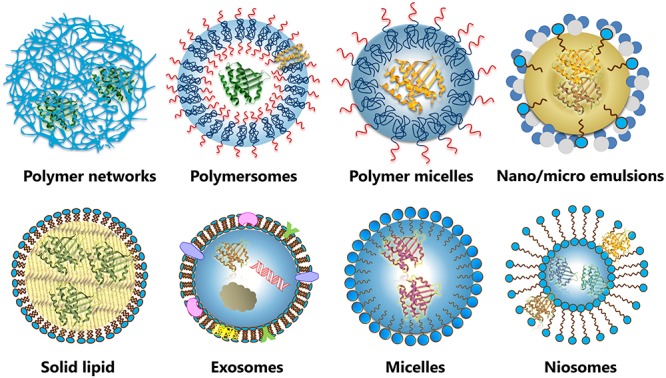
Different types of nanosized systems for protein delivery, including lipid-based, and polymer-based nanocarriers.

Beside protein-polymer conjugates and liposomes, alternative nanosized systems are under development for the delivery for therapeutic proteins (Figure 5). Advanced lipid-based and polymer-based nanocarriers show several advantages over current clinically validated systems, with the potential to overcome most of their limitations. However, the translation of nanotechnology from the bench to the market imposes several challenges (Soares et al., 2018), and many of these systems are at a development stage of proof-of-principle studies.

Lipid-based micro- and nanocarriers such as emulsions, exosomes, non-ionic surfactant vesicles, solid lipid particles and micelles and have been studied for nanoencapsulation and transport of therapeutic proteins (McClements, 2018; Liu et al., 2019).

**Emulsions** are colloidal dispersions composed of oil, water and surfactants. Depending on the formulation and manufacturing conditions, the oil-in-water or water-in-oil droplets can be small in size (microemulsion and nanoemulsions) and employed for the delivery of proteins by non-parenteral routes, such as oral and transdermal delivery (Pachioni-Vasconcelos et al., 2016; Shukla et al., 2018). They generally present high encapsulation efficiency, the manufacturing process is relatively cheap and it can easily be scaled up. However, the harsh manufacturing conditions (the use of organic phases, high mechanical forces, pressure and temperatures) may expose to the proteins to stresses and affect their activity (Tan and Danquah, 2012).

**Exosomes** are neutral extracellular vesicles (cell-derived vesicles) with a native membrane composition. These natural vesicles are involved in cell-to-cell communication and play an important role in the biomolecule transfer pathways. The similarities between exosomes and liposomes include the presence of the lipid bilayer (rich in cholesterol and diacylglycerol), the minimal toxicity, biocompatibility, the nanometric size and the internal volume where several biomolecules can be entrapped (Antimisiaris et al., 2018). The principal advantages of these nanoparticles are the high and specific organotropism and the immunocompatibility, thus representing promising vehicles for protein delivery (Hong et al., 2018). For instance, an exosomal-based delivery system for a potent antioxidant, catalase, was developed to treat Parkinson’s disease (Haney et al., 2015). Catalase was successfully encapsulated with a loading efficiency up to 26% and a sustained release was obtained *in vitro* (less that 40% in 24 h) (Haney et al., 2015). The complex preparative and purification methods and the very low isolation yields represent serious hinders to overcome (De Toro et al., 2015; Antimisiaris et al., 2018; Bunggulawa et al., 2018).

**Niosomes** are non-ionic surfactant vesicles principally composed of non-ionic surfactants and cholesterol. The particle size (from 10 nm to 20 μm) depends on the preparation method and the composition (Kaur and Kumar, 2018). Niosomes present similar advantages of liposomes in terms of ease preparation, biocompatibility, low toxicity (Kaur and Kumar, 2018; Samed et al., 2018). The main disadvantages are related to physical instability, as niosomes tend to form aggregates or fuse between themselves (Moghassemi and Hadjizadeh, 2014). However, these lipid-based carriers are in continuous development. Surfactants such as terpenoids (squalene), polysorbates, spans, alkyl oxyethylenes (usually from C12 to C18), polyoxyethylene alkyl ether and several neutral lipids have been used to obtain niosomes as nanocarriers for insulin, and peptides (Ge et al., 2019). Niosomes with sorbitan monoester were developed for vaginal delivery of insulin and tested in rats (Ning et al., 2005). These nanosystems (size 220–300 nm) were able to achieve a maximum entrapment efficiency of ∼29% and insulin release of approximately 30% in simulated vaginal fluid (Ning et al., 2005).

**Solid lipid nanoparticles** are composed of a solid lipid nucleus stabilized with a monolayer of phospholipids or surfactants. They are prepared using various lipids such as mono-, di- and triglycerides, phospholipids, fatty acids, waxes and steroids, and amphiphiles such as poloxamers and polysorbates (Geszke-Moritz and Moritz, 2016). Solid lipid nanoparticles have been extensively used for drug encapsulation, although their use for encapsulation of large biomolecules such as proteins and peptides is less conventional. A fair amount of proteins such as albumin, insulin, lysozyme, gonadorelin, antide and CyA have been encapsulated in these nanocarriers (Martins et al., 2007; Li et al., 2012). Recently, insulin-loaded solid lipid nanoparticles designed for oral delivery, formulated with an endosomal escape agent (HA2 peptides) to facilitate release, increased the absorption while maintaining the biological activity of the protein (Xu et al., 2018). Compared with subcutaneously administered free insulin, SLN administration showed a relatively slower increase in the serum insulin concentration and a significant higher relative bioavailability (3.2-fold higher than free insulin) (Xu et al., 2018).

Amphiphilic block copolymers can self-assemble into a wide range of morphologies, including micelles and polymersomes. **Polymeric micelles** are significantly more stable than surfactant-based micelles, due to their remarkably low critical micellar concentrations (10^–6^ - 10^–7^ M) and slow kinetics of dissociation, they do not undergo immediate dissolution after extreme dilution after intravenous injection (La et al., 1996). However, the encapsulation of therapeutic proteins is generally limited by the presence of the hydrophobic micellar core (Pachioni-Vasconcelos et al., 2016). Ionic-hydrophilic block copolymers have been used for the preparation of polyionic complex micelles, which may encapsulate proteins via electrostatic interactions (Insua et al., 2016).

Recently, uniform core–shell self-assembled particles, based on poly(ethylene glycol)-b-poly(l-glutamic acid) (PEG-PLE), were proposed to stabilize and to improve BDNF delivery throughout the brain (Jiang et al., 2018).

**Polymersomes** composed of block or graft amphiphilic copolymers have properties similar to those of liposomes, with the advantage of a higher membrane stability. The hydrophobic domain of the polymeric membrane can incorporate hydrophobic proteins/drugs, whereas the aqueous core can encapsulate hydrophilic proteins (Letchford and Burt, 2007). By varying block-copolymer composition, molecular weight and architecture, it is possible to tune the size, shape, membrane thickness, mechanical strength, permeability and surface chemistry for optimizing drug loading and delivery (Liu et al., 2019). Although polymerosomes are promising for protein encapsulation, further developments are required to overcome the poor encapsulation efficiency (<5% for BSA and Hb) (Lee et al., 2001). In fact, their large membrane thickness (d ≈ 8–21 nm) compared to liposomes (d ≈ 3–5 nm), represents a thermodynamic and kinetic barrier to permeability (Lee et al., 2001).

Recently, a formulation of poly(ethylene glycol)-poly(propylene sulfide) block copolymers and low molecular weight PEG was used to obtain polymersomes by a direct hydration method (O’Neil et al., 2009). Encapsulation efficiencies for ovalbumin at 37%, BSA at 19%, and bovine γ-globulin at 15%, were obtained when the proteins were included in the hydration solution (O’Neil et al., 2009).

**Polymer networks** may be used to encapsulate hydrophilic proteins within their matrix (Vermonden et al., 2012). Hydrogel nanoparticles are three-dimensional polymer networks containing a large amount of water; swelling and degradability of the hydrogel can be tuned through the choice of the type of polymer and the crosslinking density, in order to achieve an efficient protein loading and release. The polymer composition can be selected to provide stealth character, to guarantee extended plasma half-life, and to enhance targeting. For example, insulin-loaded chitosan-based hydrogel nanoparticles showed promising results for the intestinal absorption of insulin *in vivo* (Pan et al., 2002; Ma et al., 2005). Nanosized dendrimers and hyperbranched polymers have also been proposed as protein nanocarriers. Negatively charged proteins can be easily entrapped within positively charged dendrimers such as PAMAM (He et al., 2018). Dendrimer-based carriers with a hydrophobic membrane-disruptive region (aromatic motif), and a multivalent protein binding surface (guanidyl-based) was developed for the delivery of BSA, R- phycoerythrin, p53, saporin, β-galactosidase, and peptides into the cytosol of living cells (Chang et al., 2017; Liu et al., 2019). Recently, an innovative delivery system named single-protein nanocapsules (SPN) was proposed (Yan et al., 2009). In this case, polymerisable groups are covalently linked to the protein and the polymerisation occurs in an aqueous solution containing monomers and a crosslinker, resulting in each protein enfolded in a thin polymer shell. By varying the chemistry of monomers and crosslinker, it is also possible to obtain a degradable shell as well as a stimuli-responsive delivery (Lu et al., 2014; Pachioni-Vasconcelos et al., 2016). Similarly to PEGylation, limitations of SPN regards with the interference of the polymer with protein activity, because of its steric hindrance and possible conjugation of amino acids directly involved with substrate/receptor binding (Pachioni-Vasconcelos et al., 2016). Self-assembled nanostructures based on complexation with polyester nanoparticles (Choi et al., 2014; Wu et al., 2014), and layer-by-layer structures (Gu et al., 2013) have also been proposed for encapsulation and release of therapeutic proteins.

## Conclusion

Nanomedicine has already demonstrated its ability to overcome some critical limitations of protein therapeutics, and we expect to provide more examples of clinically validated technologies in the upcoming years. While protein-polymer conjugates and liposomes are well-established nanosystems with a list of therapeutically approved products, various forms of protein-loaded nanocarriers of different sizes, shapes, and compositions have been explored. The use of different nanodelivery methods and the design of nanomaterials of tunable physicochemical properties, release mechanisms and targeting strategies make these alternatives very attractive. Each of these technologies has its own advantages and disadvantages. Although some of them have successfully reached the market, the delivery of therapeutic proteins at the right concentration to the right site of action, without provoking adverse side effects, still remains a major challenge. Moreover, the development of more sophisticated nanomaterials needs a deeper understanding of their physicochemical and biological properties, and of their pharmacokinetic and pharmacodynamic effects. All these requirements, together with the need of a higher control of the manufacturing process, scale-reproducibility, and the final quality of the product, pose additional challenges in regulatory terms, which need to be addressed to achieve the maximal impact in healthcare.

## Author Contributions

All authors listed have made a substantial, direct and intellectual contribution to the work, and approved it for publication.

## Conflict of Interest

The authors declare that the research was conducted in the absence of any commercial or financial relationships that could be construed as a potential conflict of interest.
